# Supplementary material for: Evidence for the weakly coupled electron mechanism in an Anderson-Blount polar metal
Source: Nat Commun. 2019 Jul 19;10:3217. doi: 10.1038/s41467-019-11172-2 (PMC6642157; doi:10.1038/s41467-019-11172-2)
Supplement: Supplementary file 1 — Supplementary Information [file 41467_2019_11172_MOESM1_ESM.pdf]

Supplementary Information For

Evidence for the weakly coupled electron mechanism in an Anderson-Blount polar metal

N. J. Laurita et al.

Supplementary Note 1: Details of the density functional theory calculations

We performed electronic structure calculations for the $R3c$ ground state structure of $LiOsO_3$ based on density functional theory (DFT) within the revised Perdew–Burke–Ernzerhof exchange-correlation functional revised for solids[1] as implemented in the Vienna Ab initio Simulation Package[2] with the projector-augmented wave method[3] to treat the core and valence electrons using the following electronic configurations $1s^2\ 2s^1$ (Li), $6s^1\ 5d^7$ (Os), and $2s^2 2p^4$ (O), and a 550 eV plane wave cutoff. An $11 \times 11 \times 11$ Monkhorst-Pack k-point mesh[4] and Gaussian smearing of 20 meV width was used for the Brillouin zone sampling and integrations. For structure optimization we relaxed the lattice constants and the atomic positions to have forces less than 0.1 meV A^{-1}. The crystallographic parameters of the relaxed $R3c$ structure are given in Supplementary Table I while the crystal structure is shown in Figure 1a of the main text.

Atom	x	y	z	Wyckoff position	Site symmetry
Li	0.00000	0.00000	0.21443	6a	D_3
Os	0.00000	0.00000	0.00013	6a	S_6
O	0.00339	0.63367	0.25255	18b	C_2

Supplementary Table I: **Crystallographic parameters of the $R3c$ phase** Wyckoff positions are given in units of the lattice constants.

Supplementary Note 2: Demonstration that the photo-carrier dynamics are not strongly energy dependent

To ensure that the photo-carrier dynamics of $LiOsO_3$ are not strongly energy dependent, we performed time-resolved reflectivity experiments as a function of probe pulse energy. The experiment was conducted at $T = 80$ K with the pump pulse energy maintained at 1.56 eV and fluence $F = 0.5$ mJ/cm^2 while the probe pulse energy was varied with the fluence maintained at $F = 20$ μJ/cm^2. Supplementary Figure 1 displays the normalized measured reflectivity transients as a function of probe energy with fits to the bi-exponential model (dashed lines). One can see that the relaxation dynamics do not vary significantly throughout the low energy manifold of states of $LiOsO_3$.

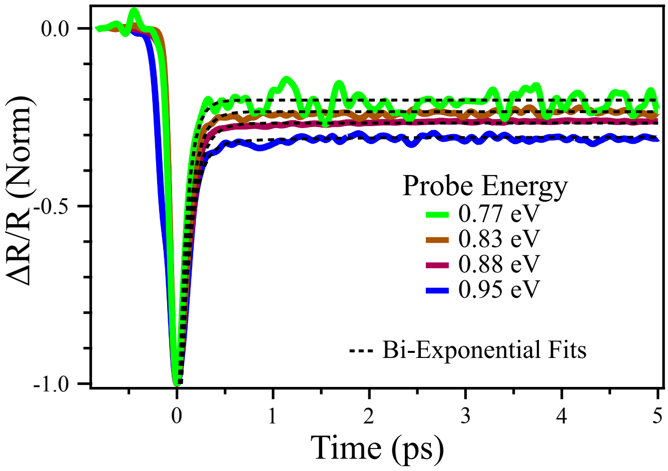

Supplementary Figure 1: **Probe energy dependent relaxation dynamics** Normalized reflectivity transients taken at $T = 80$ K as a function of probe pulse energy. Black dashed lines are fits to the bi-exponential model.

Supplementary Note 3: Demonstration that the experiment is not resolution limited

The Gaussian instrument resolution of the experiment can be determined by fitting the acquired normalized reflectivity transients with a Gaussian function for times $t < 0$ and projecting this fit to times $t > 0$. This fit represents the fastest response that the experiment can be expected to reliably measure. Supplementary Figures 2**a-f** display the measured reflectivity transients, normalized by their negative peak values, at several representative temperatures. Also shown is the Gaussian instrument resolution as a black dashed line. One can see that the instrument resolution is faster than the relaxation of the measured reflectivity transients at all temperatures, demonstrating that our measurements are not resolution limited.

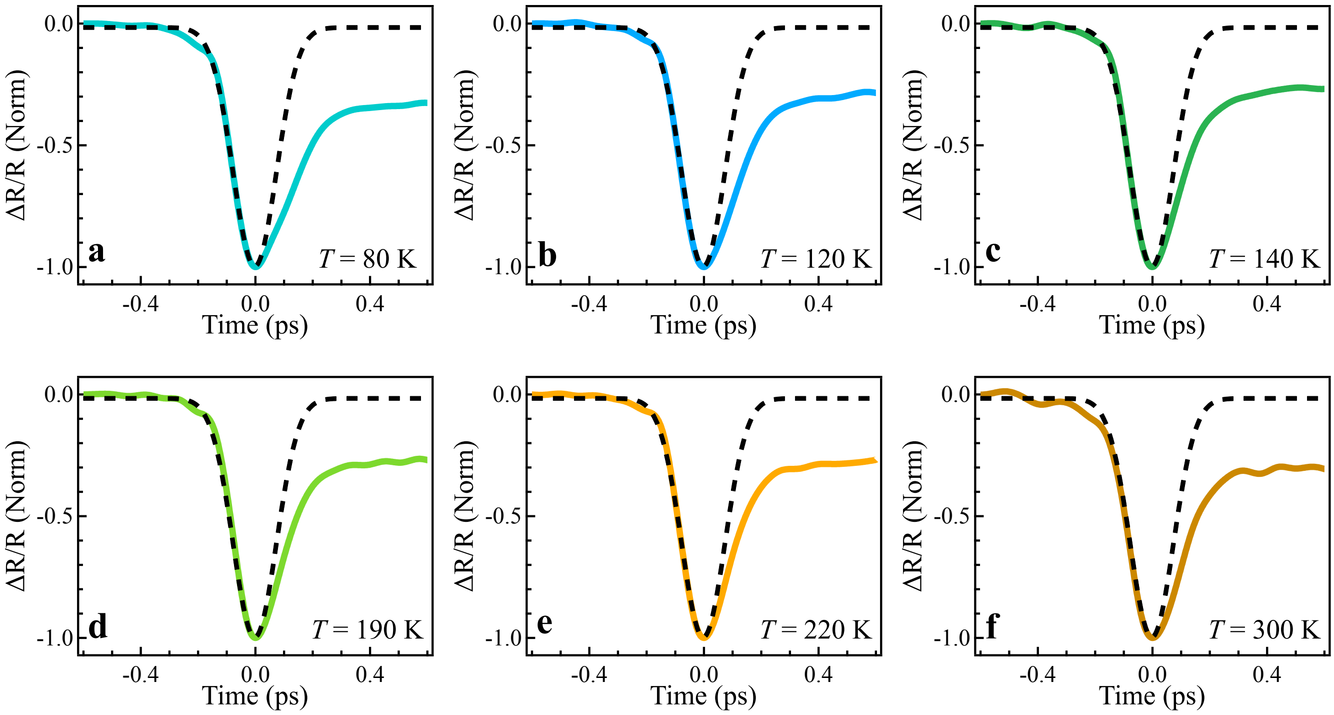

Supplementary Figure 2: **Demonstration that the experiment is not resolution limited a-f**, Normalized reflectivity transients at several representative temperatures with the Gaussian instrument resolution (black dashed line) superimposed on the data.

Supplementary Note 4: Details of the bi-exponential fits

A qualitative understanding of the temperature dependence of the measured reflectivity transients were obtained by fitting the data to a bi-exponential model given by,

$$\frac{\Delta R}{R} = A_{\mathrm{f}} \exp\left(-t/\tau_{\mathrm{f}}\right) + A_{\mathrm{s}} \exp\left(-t/\tau_{\mathrm{s}}\right) + C \tag{1}$$

where A_{f}, A_{s}, τ_{f}, and τ_{s} are the amplitudes and decay constants of the fast and slow relaxation components identified in $LiOsO_3$ respectively and C is a constant which captures heat diffusion out of the probed region of the sample. Supplementary Figure 3 displays fits of the reflectivity transients to the bi-exponential model at two representative temperatures. Experimental data is shown as black circles while bi-exponential fits of the data are shown as teal dashed lines. Also shown are each component of the fit with the corresponding fitting coefficients listed in the bottom right. Supplementary Figure 3**a** displays the data and fit at T = 300 K, where we find that a single exponential is sufficient to describe the data. However, as the temperature is reduced, the slow relaxation component grows in amplitude and must be accounted for in the fit. Supplementary Figure 3**b** shows the data and fit at T = 120 K, a temperature near where the slow relaxation is maximum.

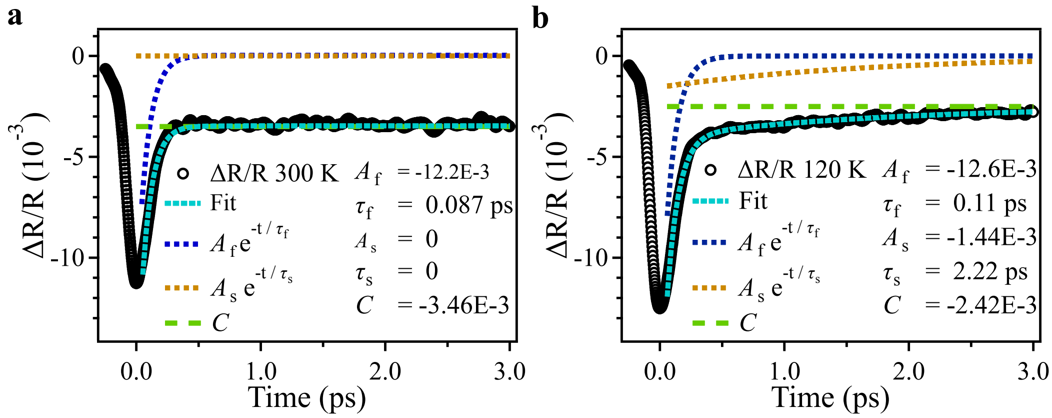

Supplementary Figure 3: **Sample fits to the bi-exponential model a,b** Reflectivity transients (black circles) at two representative temperatures, **a**, T = 300 K and **b**, T = 120 K, with the corresponding fits to the bi-exponential model (teal dashed lines). Individual components of the fits are shown as dashed lines while their corresponding fitting coefficients listed in the bottom right.

Supplementary Note 5: Calculated heat diffusion timescale

The background term C in the bi-exponential model (Supplementary Equation 1) describes heat diffusion into the bulk of the material to depths larger than the penetration depth at the probe wavelength. An estimate for this timescale is given by,

$$t_{\mathrm{c}} = \frac{\delta_{\mathrm{s}}^2 d C_{\mathrm{p}}}{M \kappa_{\mathrm{e}}} \tag{2}$$

where δ_{s} is penetration depth at the probe wavelength, d is the density, C_{p} is the lattice heat capacity, M = 245.17 g/mol is the molar mass, and κ_{e} is the thermal conductivity[5].

Aside from the lattice heat capacity[6] these quantities have yet to be reported in $LiOsO_3$. However, estimates can be made using values from comparable materials or relations with already reported quantities. The density is estimated to be $d = 4.65$ g/cm^3 from isostructural $LiNbO_3$. An estimate of the thermal conductivity is provided by the Wiedemann-Franz law, which relates κ_{e} to the resistivity as $\kappa_{\mathrm{e}} = LT_{\mathrm{e}}/\rho$, where $L = 2.44\times10^{-8}$ W Ω K^{-2} is the Lorentz number. From the reported resistivity[6], we estimate κ_{e} = 0.4 W m^{-1} K^{-1} at T = 100 K. The resistivity can also be used to calculate the penetration depth at the probe wavelength (See Supplementary Equation 6 below), which we estimate to be $\delta_{\mathrm{s}} \approx 100$ nm at T = 100 K and λ = 1350 nm.

Inserting these values into Supplementary Equation 2 results in an expected heat diffusion timescale of t_{c} = 24 ns. As this timescale is well in excess of our measurement time of 10 ps, C is well approximated by a time-independent constant.

Supplementary Note 6: Raw fluence dependent data

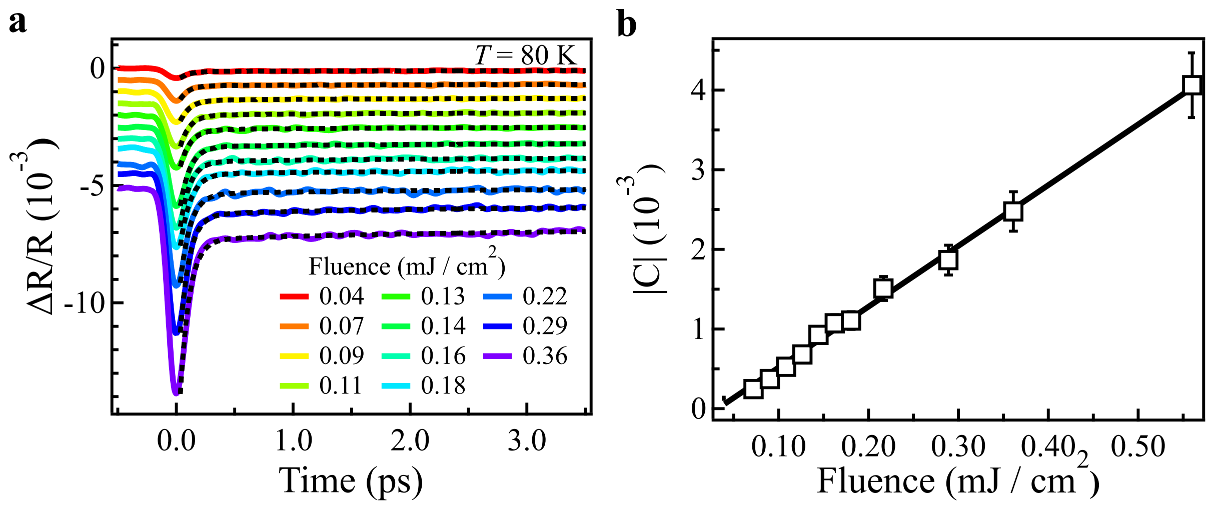

Supplementary Figure 4: **Fluence dependent relaxation dynamics a**, Reflectivity transients of $LiOsO_3$ as a function of pump fluence measured at $T = 80$ K with the pump pulse energy maintained at 1.56 eV while the probe pulse energy and fluence were maintained at 0.83 eV and $F = 20$ μJ/cm^2 respectively. Curves have been offset vertically for clarity. Black dashed lines are fits to the bi-exponential model. **b**, Fluence dependence of the constant C in the bi-exponential model. Error bars derive from the χ^2 of the bi-exponential fits.

Supplementary Note 7: Verification of rapid photocarrier thermalization

Thermalization models of transient reflectivity experiments require that the excited photocarriers initially thermalize to Fermi-Dirac distribution before thermalization with the lattice begins. To estimate the timescale for electron-electron thermalization in $LiOsO_3$, we follow the calculation outlined in Ref. 7.

In the context of Fermi liquid theory, the timescale needed for a Fermi gas to thermalize to a temperature T can be approximated by,

$$t_{\mathrm{th}} \approx \frac{1}{2K(k_{\mathrm{B}}T)^2} \tag{3}$$

where K is the electron-electron scattering rate derived from the angular averaged electron-electron scattering probability. An estimate for K via the random phase approximation is given by,

$$K = \frac{\pi^2\sqrt{3}}{128}\frac{\omega_{\mathrm{p}}}{E_{\mathrm{F}}^2} \tag{4}$$

where ω_{p} is the plasma frequency and E_{F} is the Fermi energy.

The plasma frequency of $LiOsO_3$ was measured to be $\omega_{\mathrm{p}} \approx 90$ THz in the reflectivity experiments of Lo Vecchio *et al.*[8,9]. An estimate for the charge density can then be made from ω_{p} via the Drude model of conduction in metals as $n = \epsilon_0 m^* {\omega_{\mathrm{p}}}^2/e^2$. Inserting ω_{p} into this expression, assuming $m^*/m_e = 1$, results in a carrier density of $n \approx 10^{24}$ electrons / m^3, revealing $LiOsO_3$ to be a low carrier density metal. An estimate for E_{F} can be made from the carrier density by assuming the weakly interacting Fermi gas relation,

$$E_{\mathrm{F}} = (3\pi^2)^{2/3}\frac{\hbar^2}{2m^*}n^{2/3} \tag{5}$$

which results in a Fermi energy of only $E_{\mathrm{F}} \approx 4$ meV.

With ω_{p} and E_F determined, we can now estimate the electron-electron scattering rate K. Inserting E_{F} and ω_p into Supplementary Equation 4 gives an estimated electron-electron scattering rate of $K \approx 4700$ fs^{-1} eV^{-2}. The initial electronic temperature was determined in our three temperature model to be is $T_{e,i} \approx 270$ K - 340 K (See Supplementary Equation 7 below). At these temperatures, with the estimated electron-electron scattering rate considered, Supplementary Equation 3 suggests that excited photocarriers are expected to thermalize within $t_{\mathrm{th}} \approx$ 1 fs after initial excitation, a typical timescale for thermalization in metals. As this is significantly shorter than the duration of our pump pulse, $t_{\mathrm{p}} \approx 100$ fs, excited photocarriers are expected to thermalize well before our pump pulse has left the sample, thereby satisfying the necessary initial conditions of the three-temperature model.

While the calculation presented above was performed assuming a non-interacting system, it should be noted that the true strength of electronic correlations in $LiOsO_3$ is currently an open question[8,10]. However, the calculation presented above remains valid even if electronic correlations are large, as a larger m^* would only increase K and therefore further decrease t_{th}.

Supplementary Note 8: Details of the three temperature model

Construction of the three-temperature model begins by inputting reported values for the relevant physical properties of $LiOsO_3$. Supplementary Figures 5**a-c** show the temperature dependent **a**, unit cell volume, **b**, total lattice heat capacity, and **c**, resistivity as reported by Shi *et al.*[6]. Supplementary Figure 5**d** displays the temperature dependent skin depth at our probe wavelength of $\lambda = 1350$ nm which was calculated from the reported resistivity via the relation,

$$\delta_s = \sqrt{\frac{2\rho}{\omega\mu}}\sqrt{\sqrt{1+(\rho\omega\epsilon)^2}+\rho\omega\epsilon} \tag{6}$$

where μ and ϵ are the relative permeability and permittivity of $LiOsO_3$ respectively. As the magnetic susceptibility of $LiOsO_3$ shows no signs of magnetic ordering[6], we assume $\mu = 1$ in our model. The relative permittivity was assumed to be $\epsilon \approx 10$, a typical value for conventional metals.

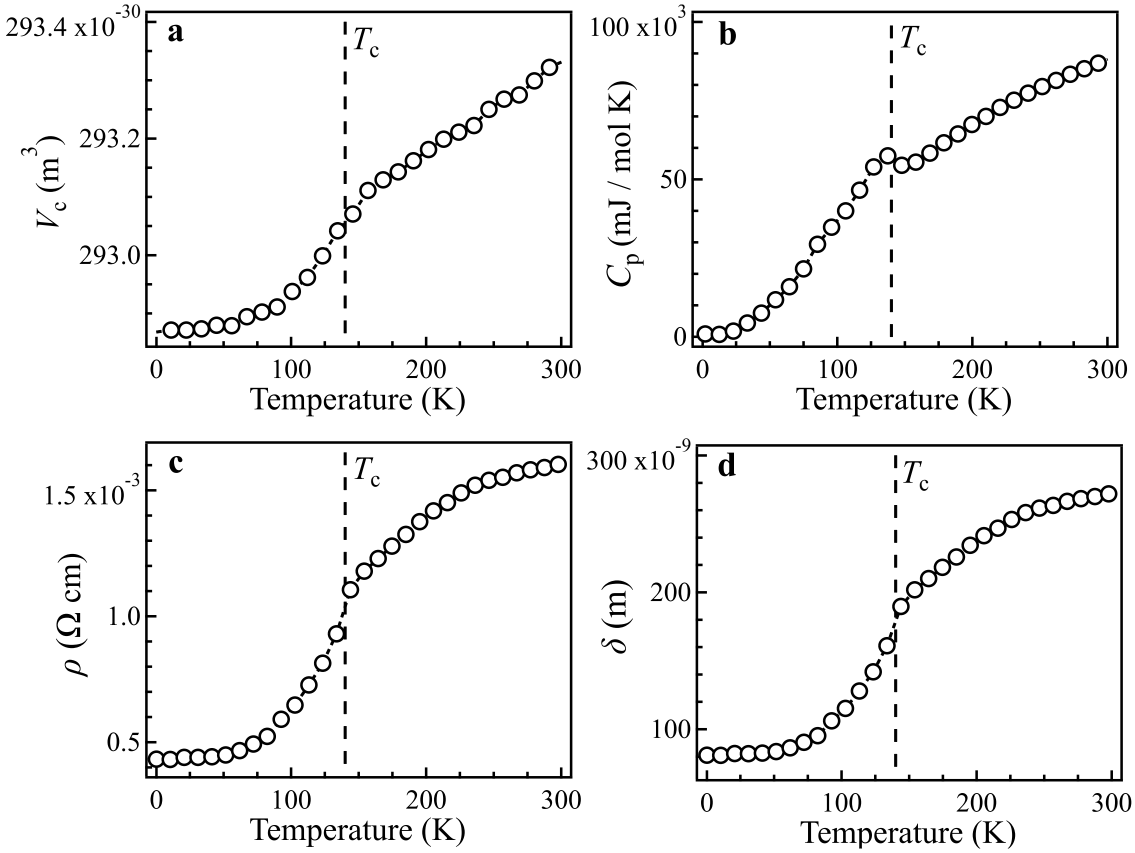

Supplementary Figure 5: **Inputs of the three temperature model** Temperature dependent **a**, unit cell volume, **b**, heat capacity, and **c**, resistivity reported by Shi. *et al.*[6]. **d**, Calculated skin depth at our probe pulse wavelength of $\lambda = 1350$ nm from the resistivity via Supplementary Equation 6.

The model assumes that the excited photocarriers strongly couple to only a subset of the phonon spectrum, which then in turn decay anharmonicly to the remainder of the phonon spectrum. The heat capacity of the excited photocarriers is assumed to be linear in temperature throughout our model and given by $C_e = \gamma T_e$ with $\gamma = 7.72$ mJ/mol K^2 as reported by Shi *et al.*[6]. The heat capacities of the strongly coupled phonons (SCPs) and the weakly coupled phonons (WCPs) are constructed by partitioning the total lattice heat capacity C_p as $C_s = \alpha C_p$ and $C_w = (1-\alpha)C_p$ respectively, where the parameter $\alpha < 1$. In constructing our model we found that the equilibrium temperature of the lattice and photocarriers was sometimes above the temperature range of the heat capacity measurement of Shi *et al.*[6]. The total lattice heat capacity was extrapolated to higher temperatures by fitting the heat capacity outside the transition region with a third order polynomial function and extending this fit to $T = 350$ K, similar to the fits presented in Figure 3(a) of Ref. 6. Above $T = 350$ K, the lattice heat capacity was assumed to be temperature independent with a value of $C_p = 9.5\times10^5$ mJ / mol K, in accordance with the law of Dulong-Petit.

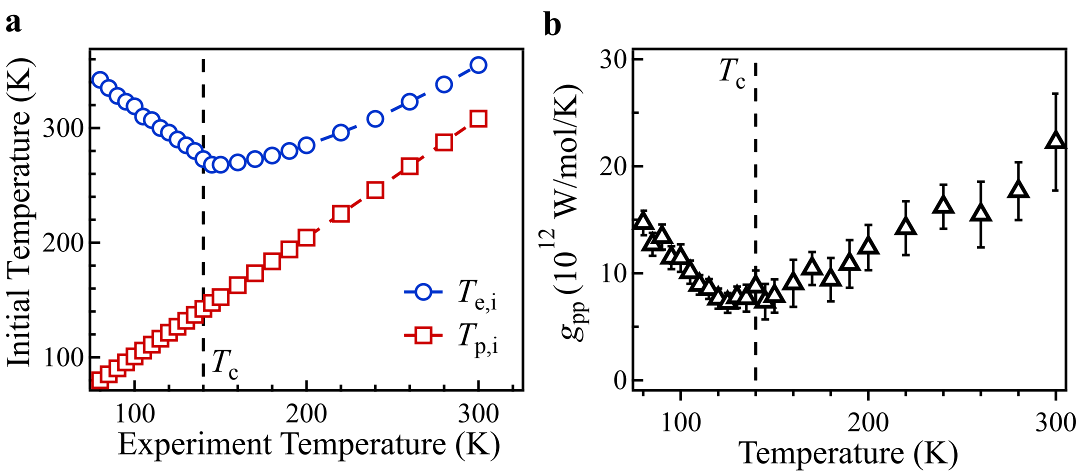

Supplementary Figure 6: **Initial temperatures and phonon-phonon coupling of the three-temperature model a**, Initial electronic (blue circles) and lattice (red squares) temperatures as a function of the initial experiment temperature in the three temperature model. **b**, The phonon-phonon coupling g_{pp} extracted from the three-temperature model. Error bars derive from the χ^2 of the three-temperature model fits.

The initial conditions of the model are the initial electronic and lattice temperatures immediately after excitation by the pump pulse. The initial lattice temperatures are simply the temperature of the experiment. The initial electronic temperatures are given by[11],

$$T_{e,i} = \frac{1}{\delta_s} \int_0^{\delta_s} \left[\sqrt{T_i^2 + \frac{2(1-R)F}{\delta_s \gamma} \exp\left(-z/\delta_s\right)} \right] dz \tag{7}$$

where $F = 0.5$ mJ /cm^2 is the pump pulse fluence, R is the reflectivity at the pump wavelength, T_i is the experiment temperature, z is the depth into the sample, and integration is performed over one penetration depth δ_s. We estimate that the reflectivity $R \approx 0.1$ and is very nearly temperature independent at our pump pulse wavelength of $\lambda = 800$ nm from the reflectivity experiments of Lo Vecchio *et al.*[8]. Supplementary Figure 6a displays the initial temperatures of the electronic (blue circles) and lattice (red squares) subsystems in our three temperature model.

Relaxation then occurs through heat exchange between the electronic and two lattice thermal baths described as captured by the equations,

$$2C_e \frac{\partial T_e}{\partial t} = -g_{ep}(T_e - T_s) + I(t,z) + \nabla \cdot [\kappa_e \nabla T_e] \tag{8}$$

$$C_s \frac{\partial T_s}{\partial t} = g_{ep}(T_e - T_s) - g_{pp}(T_s - T_w) \tag{9}$$

$$C_w \frac{\partial T_w}{\partial t} = g_{pp}(T_s - T_w) \tag{10}$$

where T_e, T_s, and T_w are the time dependent electronic and lattice temperatures respectively and g_{ep} and g_{pp} are the electron-phonon and phonon-phonon couplings. $I(t,z)$ is the laser source term given by,

$$I(t,z) = \frac{2ln(2)(1-R)F}{\pi t_p \delta_s} \exp\left[-4ln(2)(\frac{t}{t_p})^2 - \frac{z}{\delta_s}\right] \tag{11}$$

where $t_p = 100$ fs is the pulse duration. R in this case now refers to the reflectivity at our probe pulse wavelength of $\lambda = 1350$nm, which we estimate to be $R = 0.35$ from Lo Vecchio *et al.*[8]. The last term in Supplementary Equation 8 describes heat diffusion out of the probed region of the sample. An estimate for the thermal conductivity κ_e was obtained by assuming $LiOsO_3$ obeys the Wiedemann-Franz law which relates κ_e to the resistivity as $\kappa_e = LT_e/\rho$, where $L = 2.44 \times 10^{-8}$ WΩK^{-2} is the Lorentz number. Using this relation we estimate the thermal conductivity of $LiOsO_3$ to be $\kappa_e = 0.4$ W/m/K at $T_e = 100$K.

Solving the model under the experimentally dictated initial conditions gives the transient electronic and lattice temperatures, which are displayed as a function of initial experiment temperature in Supplementary Figure 7. Model

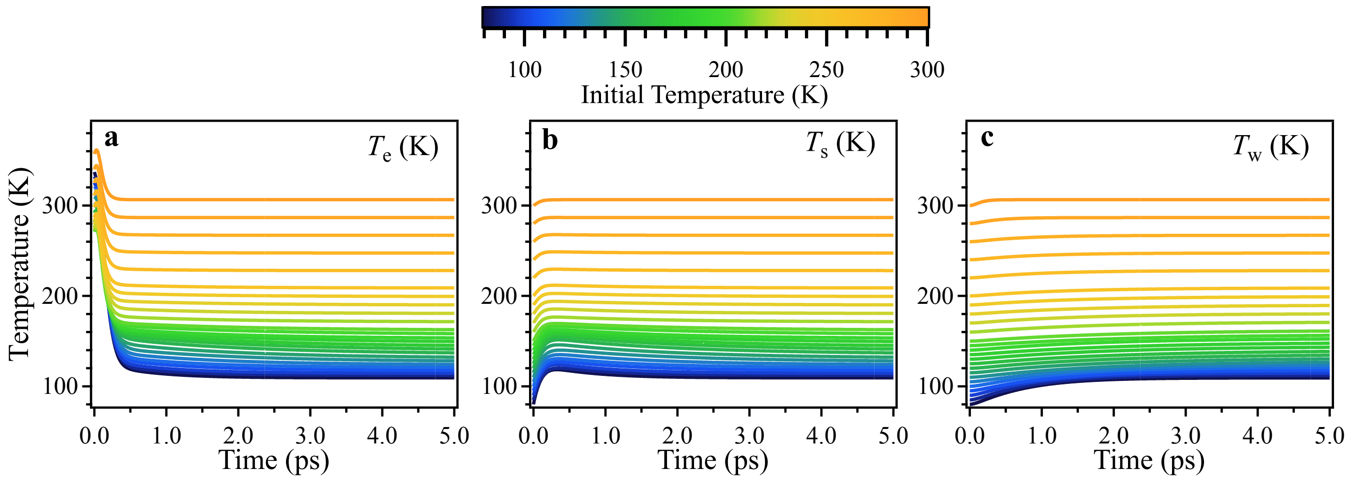

Supplementary Figure 7: **Transient temperatures of the three-temperature model** Solutions of the three-temperature model at various initial experiment temperatures as indicated by the color scale at the top. Panels **a-c** display the transient **a**, electronic T_e, **b**, strongly coupled phonon T_s, and **c**, weakly coupled phonon T_w temperatures respectively.

reflectivity transients were then constructed by assuming the conventional[11,12] linear combination of the electronic and lattice temperatures as,

$$\frac{\Delta R}{R} = aT_\mathrm{e} + b[\alpha T_\mathrm{s} + (1-\alpha)T_\mathrm{w}] \tag{12}$$

where a and b are determined by matching the initial and final values of the experimental reflectivity transients. These model reflectivity transients were then convolved with a normalized Gaussian of the form,

$$g(t) = \frac{1}{t_\mathrm{p}\sqrt{2\pi}} \exp\left[-\left(\frac{t}{\sqrt{2}t_\mathrm{p}}\right)^2\right] \tag{13}$$

where again $t_\mathrm{p} = 100$ fs is our pulse duration, to simulate the effects of our pump and probe pulses. Finally, the model reflectivity transients were then fit to the experimental data via a least squares regression algorithm with g_ep, g_pp and α as fully relaxed fitting coefficients.

Supplementary Figure 6**b** displays the phonon-phonon coupling constant g_pp extracted from the three-temperature model. The phonon-phonon coupling can be seen to decrease as the polar transition is approached, suggesting that the lattice becomes overall more harmonic as the temperature is reduced. However, g_pp increases in the polar phase. It is unclear where this increase originates but it is possible that such an increase could stem from relaxed phonon coupling selection rules in the polar phase due to broken inversion symmetry, which would permit additional coupling channels that were symmetry forbidden above the transition.

Supplementary Note 9: Comparison of the electron-phonon coupling function to the E_g phonon linewidths

First principles electron-phonon coupling theory predicts that the linewidths of coupled phonons scale with the electron-phonon coupling function as $g_{ep} \propto (\hbar\Gamma_{ph})^{1/2}$ in the zero momentum limit[13]. Such a comparison was vital in determining which phonon modes constitute the strongly coupled phonons in $LiOsO_3$. Supplementary Figure 8 shows a comparison between our extracted temperature dependent electron-phonon coupling and the scaled square roots of the linewidths of the **a**, ${}^1E_g$, **b**, ${}^2E_g$, and **c**, average E_g phonon modes as labeled by their representations in the centrosymmetric $R\bar{3}c$ space group and reported by Jin *et al.*[14]. One can see that the temperature dependence of both quantities display strong similarities. Their correlation is better observed by plotting the normalized electron-phonon coupling versus the normalized square roots of the E_g phonon linewidths, which are shown in Supplementary Figures 8**d-f**. The black dashed lines represent the limit of perfect correlation $g_{ep} \propto \Gamma_{ph}^{1/2}$. These plots demonstrate the strong correlation between these two quantities.

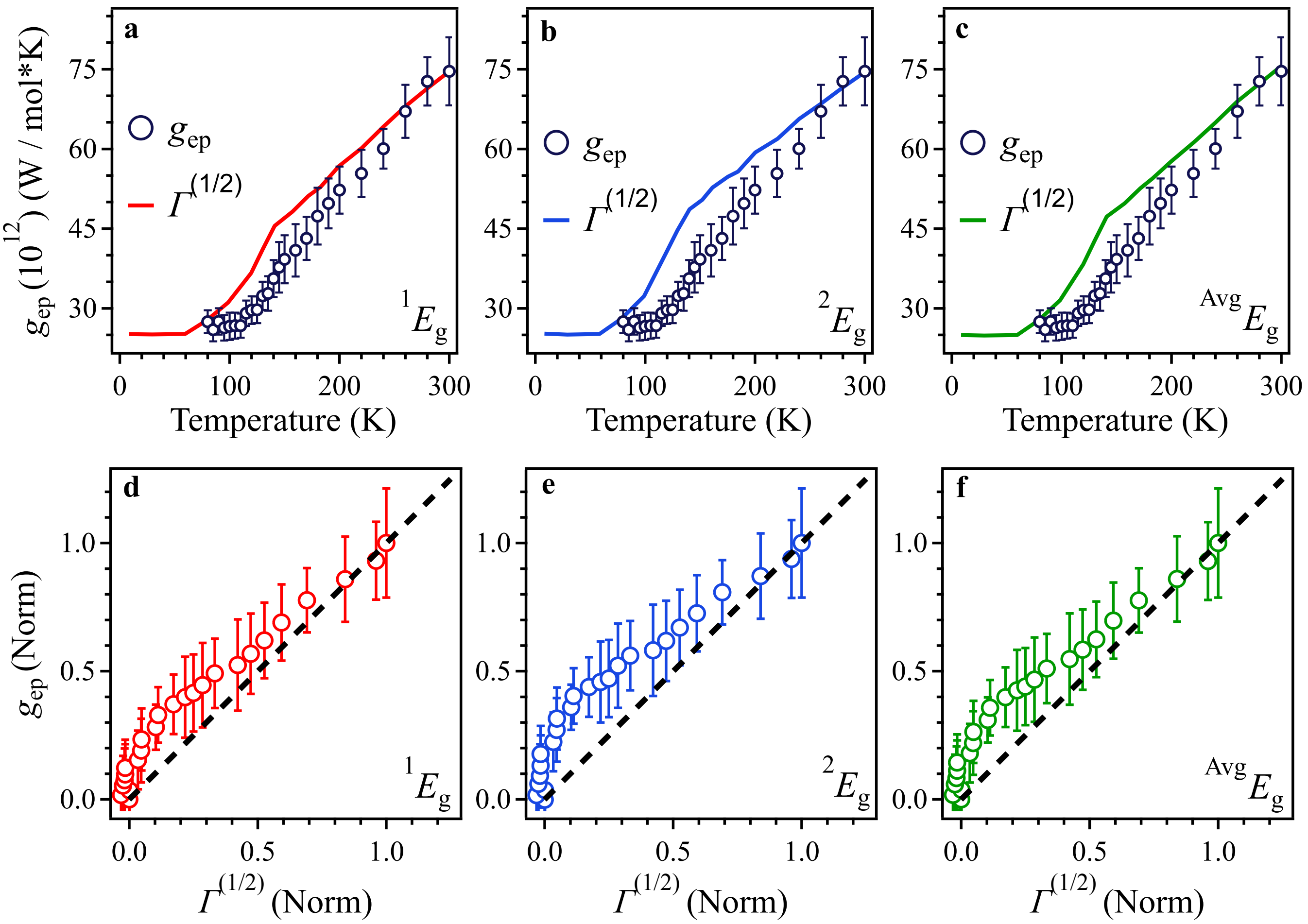

Supplementary Figure 8: **Identification of the strongly coupled phonons** The temperature dependent electron-phonon coupling function g_{ep} extracted from our three-temperature model plotted with the scaled square roots of the **a**, ${}^1E_g$, **b**, ${}^2E_g$, and **c**, average E_g phonon linewidths reported via Raman spectroscopy[14]. **d-f**, Plots of the normalized g_{ep} versus the normalized square roots of the E_g phonon linewidths showing a strong correlation between these two quantities. Black dashed lines represent the limit of perfect correlation $g_{ep} \propto \Gamma_{ph}^{1/2}$. Error bars derive from the χ^2 of the three-temperature model fits.

Supplementary Note 10: Details of the displacive soft mode model of the weakly coupled heat capacity

From the three-temperature model analysis, we concluded that the itinerant electrons of $LiOsO_3$ are decoupled from a significant portion of the total lattice heat capacity. We argued that at temperatures $T < 250$ K, at which point the other optical phonon modes are expected to be largely frozen out, the decoupled heat capacity can be mostly attributed to that of the A_{2u} polar mode. That is,

$$1 - \alpha \approx C_{A_{2u}}/C_{\rm p} \tag{14}$$

where $C_{A_{2u}}$ is the heat capacity of the A_{2u} polar mode and $C_{\rm p}$ is the temperature dependent total lattice heat capacity of $LiOsO_3$ as reported by Shi *et al.*[6].

To model 1 - α, the polar mode was treated as an Einstein phonon whose temperature dependent heat capacity is given by

$$C_{A_{2u}} = A(\frac{\hbar\omega}{k_{\rm B}T})\frac{e^{\hbar\omega/k_{\rm B}T}}{[e^{\hbar\omega/k_{\rm B}T}-1]^2} \tag{15}$$

where A is a proportionality constant and $\hbar\omega$ is the energy of the polar mode. Supplementary Equation 15 was inserted into Supplementary Equation 14 and fit to 1 - α with the phonon frequency as the only free parameter. The proportionality constant A was chosen such that the polar mode energy softened to zero at the transition as expected for a displacive mode. Fitting the data in this fashion allows one to extract the temperature dependent heat capacity and frequency of the polar mode which was shown in the inset of Figure 4d of the main text.

Supplementary Note 11: Phonon dispersions of the polar R3c phase

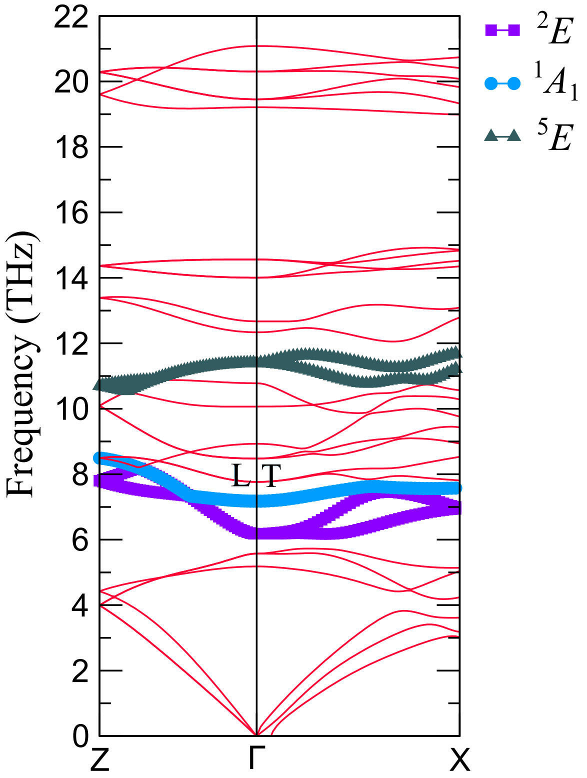

Supplementary Figure 9: **Phonon dispersions of the R3c phase of $LiOsO_3$** The polar A_{2u} mode and the doubly degenerate ^1E_g and ^2E_g modes, which transform as $^1A_1$, ^2E, and ^5E respectively in the polar phase in accordance with the notation of[14], are highlighted. Only the polar mode can be defined as longitudinal (L) or transverse (T) in the Brillouin zone, as the other modes exhibit both longitudinal and transverse components throughout the Brillouin zone.

Supplementary References

[1] J. P. Perdew *et al.* Restoring the density-gradient expansion for exchange in solids. Phys. Rev. Lett. **100**, 136406 (2008).

[2] G. Kresse and J. Furthmüller. Efficiency of ab-initio total energy calculations for metals and semiconductors using a plane-wave basis set. Computational Materials Science **6**, 15 (1996).

[3] P. E. Blöchl, O. Jepsen, and O. K. Anderson. Improved tetrahedron method for Brillouin-zone integrations. Phys. Rev. B. **49**, 16223 (1994).

[4] H. J. Monkhorst and J. D. Pack. Special points for Brillouin-zone integrations. Phys. Rev. B. **13**, 5188 (1976).

[5] D. Hsieh *et al.* Observation of a metal-to-insulator transition with both Mott-Hubbard and Slater characteristics in Sr_2IrO_4 from time-resolved photocarrier dynamics. Phys. Rev. B. **86**, 035128 (2012).

[6] Y. Shi *et al.* A ferroelectric-like structural transition in a metal. Nature Materials **12**, 1024-1027 (2013).

[7] R. H. M. Groeneveld, R. Sprik, and A. Lagendijk. Femtosecond spectroscopy of electron-electron and electron-phonon energy relaxation in Ag and Au. Phys. Rev. B. **51**, 11433 (1995).

[8] I. Lo Vecchio. *et al.* Electronic correlations in the ferroelectric metal $LiOsO_3$. Phys. Rev. B. **93**, 161113 (2016).

[9] Private Communication.

[10] H. M. Lin, *et al.* Metallic ferroelectricity induced by anisotropic unscreened Coulomb interaction in $LiOsO_3$. Phys. Rev. B. **91**, 064104 (2015).

[11] B. Mansart, *et al.* Ultrafast transient response and electron-phonon coupling in the iron-pnictide superconductor $Ba(Fe_{1-x}Co_x)_2As_2$. Phys. Rev. B. **82**, 024513 (2010).

[12] S. D. Brorson, *et al.* Femtosecond room temperature measurement of the electron-phonon coupling constant γ in metallic superconductors. Phys. Rev. Lett. **64**, 2172 (1990).

[13] F. Giustino. Electron-phonon interactions from first principles. Rev. Mod. Phys. **89**, 015003 (2017).

[14] F. Jin. *et al.* Raman phonons in the ferroelectric-like metal $LiOsO_3$. Phys. Rev. B. **93**, 064303 (2016).
